# Effect of Danhong injection on prognosis and inflammatory factor expression in patients with acute coronary syndrome during the perioperative period of percutaneous coronary intervention: A systematic review and meta-analysis

**DOI:** 10.3389/fcvm.2022.1029387

**Published:** 2022-11-21

**Authors:** Yuxuan Li, Dong Li, Wujiao Wang, Xingxing Li, Peng Li, Yuanyuan Zhang, Qian Lin, Yan Li

**Affiliations:** ^1^Second Clinical School of Medicine, Beijing University of Chinese Medicine, Beijing, China; ^2^Department of Cardiology, Dongfang Hospital, Beijing University of Chinese Medicine, Beijing, China; ^3^Department of Cardiology, Dongzhimen Hospital, Beijing University of Chinese Medicine, Beijing, China

**Keywords:** Danhong injection (DHI), perioperative period, acute coronary syndrome (ACS), prognosis, inflammatory factor expression, percutaneous coronary intervention (PCI)

## Abstract

**Objectives:**

In China, Danhong injection (DHI) is recommended by expert consensus and is widely used in the perioperative management of patients with acute coronary syndrome (ACS). This study investigates the effect of perioperative DHI administration and the timing of DHI administration on patients with ACS undergoing percutaneous coronary intervention (PCI) by analyzing the prognosis and anti-inflammatory effects. This article summarizes the most up-to-date clinical evidence on DHI, and in this study, we assesses treatment efficacy of DHI in patients with ACS.

**Methods:**

A total of seven databases (PubMed, Embase, Cochrane Library, SINOMED, CNKI, Wanfang, and VIP) were searched from the time of their inception to 1 July 2022. Clinical randomized controlled trials (RCTs) of DHI combined with PCI for the treatment of ACS were included. RCT quality was assessed using the Cochrane Handbook risk-of-bias tool, and STATA 17.0 was used for meta-analysis.

**Results:**

In total, 33 studies including 3,458 patients with ACS undergoing PCI were included in the meta-analysis. Compared with conventional therapy alone, the combination of DHI and conventional therapy significantly decreased the incidence of major adverse cardiovascular events (MACEs; *P*<0.001) and improved the reperfusion rate (*P* < 0.001). Serum high-sensitivity C-reactive protein (hs-CRP) and interleukin (IL)-6 levels were substantially reduced in the test group (*P*<0.001). In addition, the plasma levels of myocardial injury markers and cardiac troponin T (cTnT) declined significantly (*P* < 0.01). Compared with the control group, DHI improved the left ventricular ejection fraction (LVEF; *P* < 0.001) and reduced B-type natriuretic peptide (BNP; *P* < 0.001) levels. Subgroups were established based on different timings of DHI administration: preoperative, intraoperative, and postoperative groups. The results showed that the incidence of MACEs and the reperfusion rate did not differ between the groups. Among the subgroups, the postoperative group exhibited significantly lower levels of BNP, hs-CRP, and IL-6 serum and a significantly higher level of LVEF (*P* < 0.05).

**Conclusion:**

The combination of DHI and conventional therapy results in a better therapeutic effect than that observed with conventional therapy alone in patients with ACS. To improve treatment efficacy, postoperative initiation of DHI is recommended as a standard treatment. Further research is needed to confirm these results.

**Systematic review registration:**

Identifier: CRD42022344830.

## Introduction

Acute coronary syndrome (ACS) is caused by the rupture of atherosclerotic plaque and subsequent thrombosis, resulting in unstable angina, non-ST segment elevation myocardial infarction, and ST segment elevation myocardial infarction (1, 2). In China, the incidence of coronary heart disease (CHD) and associated mortality rates is increasing annually (3). ACS is the most extreme type of CHD. Percutaneous coronary intervention (PCI) has an immediate effect on the revascularization of the infarct-related artery, and it may be more effective in restoring myocardial perfusion, reducing the incidence of myocardial ischemia or infarction, and improving clinical outcomes. PCI is widely used for the treatment of ACS (4). However, PCI may be complicated by no reflow, slow coronary flow, diverse arrhythmias, myocardial ischemia–reperfusion injuries (MIRIs), and in-stent restenosis (ISR). MIRI seriously affects patients' heart function and prognosis. Therefore, these complications of PCI cannot be ignored.

As a complementary or adjuvant therapy, DHI is a standardized traditional Chinese medicine (TCM) product. The main active ingredients are protocatechuic aldehyde, tanshinone, salvianolic acid, and catechin (5). Based on the TCM theory, the pathogenesis of CHD is closely related to stagnant blood, while DHI promotes blood flow and resolves the blood stasis. Modern pharmacological studies have reported that DHI promotes multiple pharmacological activities that have anti-thrombotic, anti-platelet aggregate, anti-inflammatory, hypolipidemic, anti-oxidative damage, and pro-human microcirculation effects (6). In clinical practice, DHI has been used to treat cardiovascular diseases and to reduce the incidence of major adverse cardiovascular events (MACEs), myocardial necrosis marker levels, and inflammatory factor levels (7–10).

A previous meta-analysis reported that DHI combined with conventional therapy for the treatment of patients with ACS improved the total efficacy rate and decreased the incidence of MACEs after PCI (11). However, it did not measure indicators such as myocardial injury or analyze the effect of the timing of DHI.

Therefore, this systemic review and meta-analysis summarizes the results of more recent RCTs regarding DHI. The efficacy of DHI in patients with ACS undergoing PCI and the effect of the timing of DHI on the incidence of MACEs and myocardial injury and inflammatory biomarker levels are assessed to provide clinical evidence regarding DHI.

## Materials and methods

This analysis followed the PRISMA guidelines (12), and the review protocol was registered with PROSPERO (CRD42022344830).

### Search strategy

For this study, seven databases (PubMed, Embase, Cochrane Library, SINOMED, CNKI, Wanfang, and VIP) were searched from their inception to 1 July 2022, using the following subject terms: “percutaneous coronary intervention,” “Danhong injection,” “acute coronary syndrome,” “myocardial infarction,” “unstable anginas,” “percutaneous coronary intervention,” and “randomized controlled trial.” The search terms were changed according to databases and languages. Language restrictions were not applied for included studies. The different databases used a corresponding combination of subject words, free words, and keywords. In total, two researchers (YXL and YL) independently evaluated the eligibility of the retrieved studies. A third researcher (DL) was consulted in case of disagreement. The bibliography of each article was manually searched for additional studies.

### Inclusion and exclusion criteria

The prespecified eligibility criteria were as follows: (a) RCTs including patients with ST elevation myocardial infarction or unstable angina/non-ST elevation myocardial infarction, as defined by the European Society of Cardiology guidelines, and undergoing PCI were included in the meta-analysis; (b) all studies including a control group undergoing conventional therapy and a test group undergoing DHI combined with conventional treatment; and (c) studies reporting at least one of the following findings or outcomes: MACEs, thrombolysis in myocardial infarction (TIMI) flow grade, ST segment resolution (STR), high-sensitivity C-reactive protein (hs-CRP), interleukin (IL)-6, creatine kinase (CK), CK–myocardial band (MB), cardiac troponin T (cTnT), left ventricular ejection fraction (LVEF), or brain natriuretic peptide (BNP).

The exclusion criteria were as follows: (a) duplicate studies, (b) studies in which other TCMs were used in control or experimental groups, and (c) case reports, narrative reviews, meta-analyses, systematic literature reviews, observational studies, animal studies, or *in vitro* studies.

### Data extraction

In this study, two researchers (YXL and YL) independently extracted data from each study, including the first author, year of publication, participant characteristics, sample size, intervention, duration of intervention, and outcome assessment, and any differences were resolved *via* discussion.

### Quality assessment

The quality of the included studies was assessed following the Cochrane Handbook of Systematic Review. Random sequence generation, assignment confounding, blinding of participants and hospital staff, blind outcome assessment, incomplete outcome data, selective reporting, and other sources of bias were considered in the quality assessment. The results were cross-checked by the same two researchers (YXL and YL), and any disagreements were resolved *via* discussion.

### Data synthesis and statistical analysis

STATA 17.0 was used for the meta-analysis (13). Data are presented as risk ratios (RRs) and standardized mean differences (SMDs) with 95% confidence intervals (CIs). Potential heterogeneity was assessed using Cochran Q and *I*^2^ statistical tests. A fixed-effects model was used to compare data from studies with low heterogeneity (14), whereas a random-effects model was used to compare data from studies with high heterogeneity (*P* < 0.05, *I*^2^ > 50%). Subgroup, sensitivity, and meta-regression analyses were used to examine heterogeneity between the outcomes. The potential of a publication bias was assessed using Egger's and Begg's tests.

## Results

### Literature search and screening

**Figure 1:**
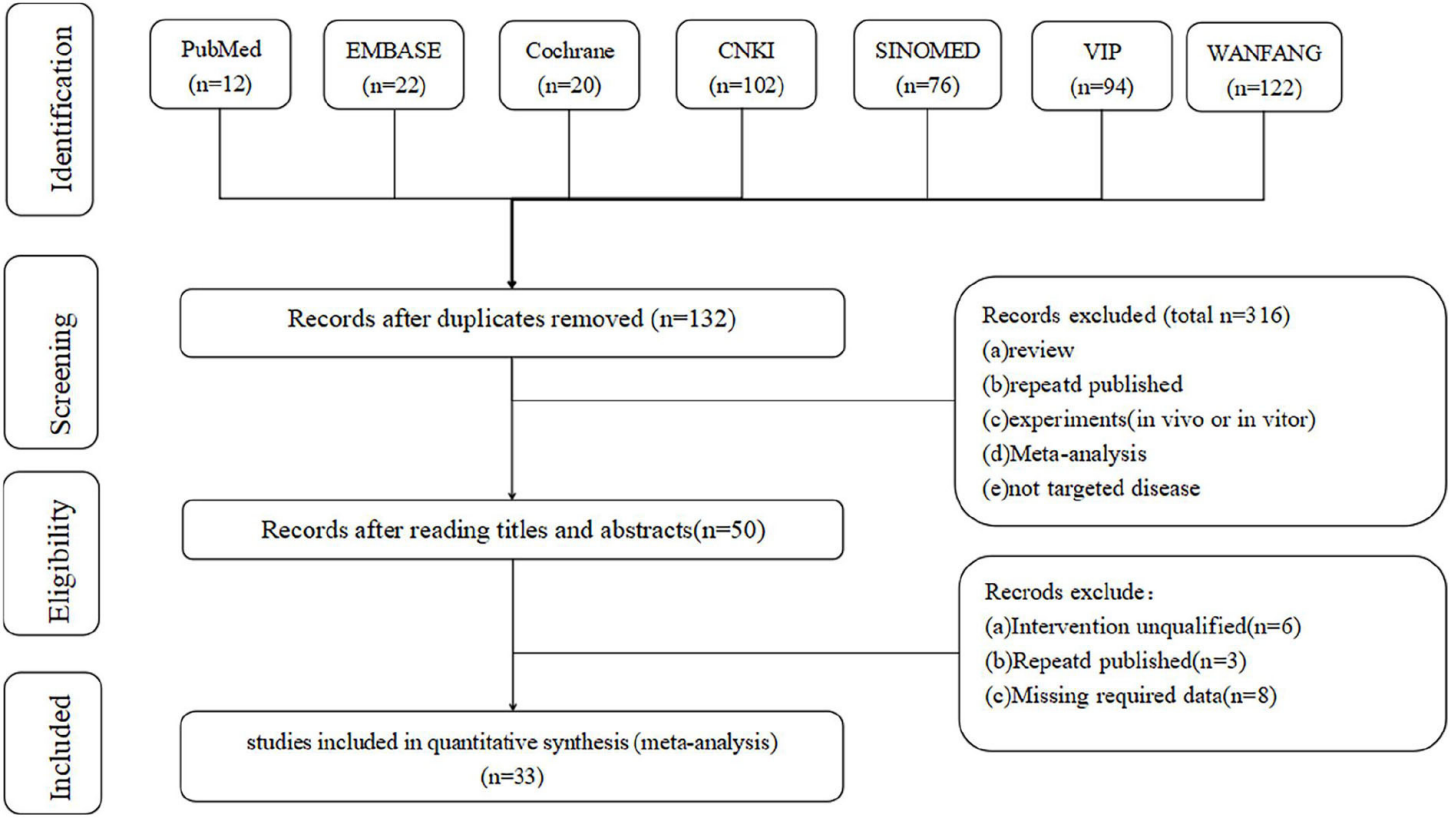
Detailed process flowchart of the study selection.

First, the database search identified 448 articles for evaluation (Wanfang: 120 articles; CNKI: 102 articles; VIP: 93 articles; SINOMED: 76 articles; Embase: 22 articles; Cochrane Library: 20 articles; and PubMed: 12 articles). Subsequently, 316 duplicate records were identified and removed. After screening titles and abstracts, 82 articles met the exclusion criteria. Finally, 33 articles (7–10, 15–43) were included in the meta-analysis (Figure 1).

### Study characteristics and quality assessment

The 33 studies included in this meta-analysis were published between 2007 and 2022 (Table 1) and were conducted in China. Among them, two studies were published in English (35, 42) and 31 studies were in Chinese. A total of 3,458 patients with ACS who underwent PCI were enrolled, among whom 1,722—control group—patients received a PCI-based conventional treatment, including medicines for anti-platelet aggregation, anti-coagulation, lipid lowering and plaque stabilization, and inhibition of ventricular remodeling, and the remaining 1,736—test group—patients received DHI and the conventional treatment. A DHI dose of 20–40 mL was used, administered by intravenous drip or injection. DHI was administered before (preoperatively), during (intraoperatively), or after (postoperatively) PCI. The treatment duration was 7–14 days. The outcome indicators of response prognosis observed in the included studies were MACEs, STR, and ISR, and the inflammatory factors were hs-CRP, tumor necrosis factor (TNF)-α, IL-6, IL-10, and matrix metalloproteinase (MMP)-9. Markers of myocardial injury (CK, CK-MB, and cTnT) and cardiac function (LVEF and BNP) were also reported.

**Table 1:** Study characteristics.

**References**	**Disease**	**Sample** **size (T/C)**	**Participants** **(Male/Female)**	**Age (years)**		**Intervention**		**DHI** **(dosage and** **method)**	**Duration**	**Intervention** **time**	**OutCOME**
				**T**	**C**	**T**	**C**				
Feng et al. (15)	ACS	91(46/45)	66/ 25	67.2 ± 16.2	65.6 ± 17.3	DHI+PCI+CT	PCI+CT	40 ml qd ivgtt	28 days	Preoperative	MACEs+hs-CRP+TC+TG+LDL+ET-1+Fg
Gao et al. (16)	STEMI	61(31/30)	38/ 23	60.1 ± 10.6	59.8 ± 7.6	DHI+PCI+CT	PCI+CT	40 ml qd ivgtt	14 days	Postoperative	MACEs+hs-CRP
Chen et al. (17)	ACS	100(50/50)	62/ 38	63.1 ± 9.7	67.5 ± 8.8	DHI+PCI+CT	PCI+CT	40 ml qd ivgtt	14 days	Postoperative	hs-CRP+CD62p+GP+FIB-C
Chen et al. (18)	STEMI	58(29/29)	43/ 16	61.9 ± 5.2	65.2 ± 4.5	DHI+PCI+CT	PCI+CT	20 ml qd ivgtt	14 days	Postoperative	STR+hsCRP+ET-1+LVEF
Zhao et al. (19)	ACS	70(36/34)	37/ 33	54.00 ± 9.00	54.00 ± 9.00	DHI+PCI+DAAT +PS+UFH	PCI+DAAT+PS +UFH	40 ml qd ivgtt	14 days	Postoperative	hs-CRP+ET-1+sP-sel
Zhang and Zhang (20)	ACS	68(34/34)	37/ 31	55.7 ± 7.4	54.5 ± 8.2	DHI+PCI+CT	PCI+CT	40 ml qd ivgtt	14 days	Postoperative	STR+hs-CRP+CD62p+ET-1
Wang et al. (21)	STEMI	60(30/30)	44/ 16	65.22 ± 7.54	63.61 ± 8.21	DHI+PCI+DAAT	PCI+DAAT	20 ml iv+20 ml qd ivgtt	st	intraoperative	STR+IL-6
Cui and Wang (22)	AMI	180(90/90)	106/ 74	72.1 ± 5.8	72.3 ± 5.8	DHI+PCI+CT	PCI+CT	30 ml qd ivgtt	10 days	Postoperative	Clinical efficiency+hs-CRP+SOD
Dong (10)	UA	120(60/60)	90/ 30	58.3 ± 10.2	56.8 ± 8.6	DHI+PCI+DAAT +PS+a-gent	PCI+DAAT +PS+a-gent	40 ml qd ivgtt	7 days	intraoperative	MACEs+TIMI+hsCRP+ IL-6+CK-MB+cTnT+ SOD+Vwf+sICAM-1 +LVWM
Qin et al. (9)	AMI	112(56/56)	61/ 51	52.31 ± 11.24	55.12 ± 10.52	DHI+PCI+DAAT +PS+ARB/ACEI+β blockers +UFH	PCI+DAAT+PS +ARB/ACEI+β blockers +UFH	40 ml qd ivgtt	7 days	Postoperative	hs-CRP+CK-MB+cTnT+SOD+BNP +LVEF
Chen et al. (8)	ACS	120(60/60)	65/ 55	61.38 ± 8.63	61.47 ± 9.38	DHI+PCI+CT	PCI+CT	40 ml qd ivgtt	14 days	Postoperative	MACEs+ISR+LDL-C+TC +TG+CD6P+PAGT+ PADT+LVEF
Guo et al. (7)	ACS	125(62/63)	69/ 56	62.1 ± 10.6	61.5 ± 10.3	DHI+PCI+CT+CE	PCI+CT+CE	40 ml qd ivgtt	14 days	Postoperative	MACEs+CRP+IL-1+TNF-α+vWF+FMD+ ET-1+NO
Guo et al. (23)	ACS	78(38/40)	45/ 33	60.1 ± 10.6	61.6 ± 11.2	DHI+PCI+CT	PCI+CT	40 ml qd ivgtt	14 days	Postoperative	hs CRP+ICAM-1+VCAM-1
Jia et al. (24)	AMI	120(60/60)	75/ 45	62.23 ± 11. 26	64.56 ± 12. 85	DHI+PCI+CE	PCI+CE	20mg st ivgtt	st	intraoperative	TIMI+CRP
Xu et al. (25)	AMI	71(36/35)	49/ 22	65 ± 13	63 ± 11	DHI+PCI+CT	PCI+CT	40 ml qd ivgtt	14 days	Postoperative	MACEs+STR+CK+CK-MB+cTnT+ET-1+BNP+LVEF
Yang et al. (26)	STEMI	57(28/29)	30/ 27	64 ± 12. 3	65 ± 11. 7	DHI+PCI+CT	PCI+CT	40 ml qd ivgtt	10 days	Postoperative	MACEs+STR+TIMI+CK +CK-MB+cTnT+ET-1 +IRA +BNP+LVEF
Zheng et al. (27)	STEMI	300(150/150)	186/ 114	61.7 ± 7.4		DHI+PCI+DAAT +UFH	PCI+DAAT +UFH	30 ml qd ivgtt	10 days	Postoperative	STR+IL-17+IL-6+MIS+LVEF
Zhou et al. (28)	UA	100(50/50)	70/ 30	58.0 ± 9.2		DHI+PCI+CT	PCI+CT	40 ml qd ivgtt	7 days	Preoperative	Clinical efficiency+hs-CRP+IL-6+cTnT
Liu et al. (29)	ACS	104(52/52)	55/ 49	58.73 ± 8.45	59.21 ± 8.57	DHI+PCI+DAAT +UFH	PCI+DAAT +UFH	40 ml qd ivgtt	14 days	Postoperative	Vwf+ET-1+NTG+NO +FMD+pentraxin-3+ IL-18+IL-18/IL-10+ LpPLA2+IL-10+BNP +LVEF
Zhang et al. (30)	ACS	100(50/50)	67/ 33	71.26 ± 4.82	68.28 ± 4.88	DHI+PCI+CT	PCI+CT	40 ml qd ivgtt	14 days	Postoperative	hs-CRP+ET-1+IL-6+Vwf+NO+FMD
Zeng et al. (33)	STEMI	120(60/60)	64/ 56	65.13 ± 2.38	64.38 ± 2.12	DHI+PCI+CT	PCI+CT	40 ml qd ivgtt	14 days	Postoperative	MACEs+Clinical efficiency+IL-6+IL-17+LVEF+MIS
Liu et al. (31)	NSTEMI	180(90/90)	NR	NR	NR	DHI+PCI+DAAT +UFH	PCI+DAAT +UFH	20 ml qd ivgtt	14 days	Postoperative	hs-CRP+Clinical efficiency+ET+LVEF
Wu et al. (32)	STEMI	80(44/36)	NR	NR	NR	DHI+PCI+CT	PCI+CT	4 ml iv+20 ml st ivgtt	st	intraoperative	STR+MMP-9+CRP+IL-6
Qin et al. (34)	AMI	126(63/63)	62/ 64	63.98 ± 1.25	63.41 ± 1.16	DHI+PCI+CT	PCI+CT	4 ml iv+20 ml ivgtt st	st	intraoperative	MACEs+TIMI+IL-6+Cys-C+Hcy+LVEF
You et al. (35)	STEMI	110(57/53)	95/ 15	56.8 ± 8.9	55.4 ± 9.5	DHI+PCI+CT	PCI+CT	40 ml qd ivgtt	4-6 days	Preoperative	MACEs+CK-MB+cTnT+MIS+LVEF
Hu (36)	AMI	86(43/43)	65/ 21	50.28 ± 0.43	50.62 ± 0.53	DHI+PCI+CT	PCI+CT	30 ml qd ivgtt	14 days	Postoperative	Clinical efficiency+CRP+IL-6+FIB+D-Dimer+CD63+CD62P+ SOD+MDA
Lv (37)	AMI	100(50/50)	62/ 38	60 ± 5.8	59 ± 6	DHI+PCI+CT	PCI+CT	40 ml qd ivgtt	14 days	Postoperative	MACEs+STR+hs-CRP+cTnT+CK-MB+NT-proBNP
Wen-long (42)	UA	78(39/39)	58/ 20	61.03 ± 9.03	60.74 ± 10.82	DHI+PCI+CT	PCI+CT	40 ml qd ivgtt	7 days	intraoperative	MACEs+CK+CK-MB+cTnT+FFR+IMR
Chen et al. (38)	STEMI	93	56/ 37	62.9 ± 9.5	63.2 ± 8.5	DHI+PCI+CT	PCI+CT	40 ml st ivgtt	st	intraoperative	MACEs+TIMI+STR+ Arrhythmia+hs-CRP+CK+CK-MB+CTNI+FIB+LDH +NT-proBNP
Cui et al. (39)	AMI	90	46/ 44	57.53 ± 3.35	56.35 ± 3.23	DHI+PCI+CT	PCI+CT	30 ml qd ivgtt	60 days	Postoperative	MACEs+STR+ISR+ Clinical efficiency+hsCRP+MMP-9+TNF-α+ET-1
Feng (40)	STEMI	157	82/ 76	60.19 ± 1.38	60.25 ± 1.21	DHI+PCI+CT	PCI+CT	20 ml qd ivgtt	7 days	Postoperative	ANGPTL4+Sst2+LVEF
Niu et al. (41)	UA	61	NR	NR	NR	DHI+PCI+CT	PCI+CT	40 ml bid ivgtt	7 days	Preoperative	MACEs+CK-MB+Metabolome
Li et al. (43)	AMI	82	49/ 33	62.5 ± 4.6	62.3 ± 4.5	DHI+PCI+CT	PCI+CT	20 ml qd/20 ml bid	7 days	Postoperative	MACEs+Clinical efficiency+hs-CRP+TNF-α+IL-6+LEVF

T, trial group; C, control group; NR, no report; ACS, acute coronary syndrome; UA, unstable angina; AMI, acute myocardial infarction; STEMI, ST segment elevation myocardial infarction; CT, conventional therapy, no details were given; DHI, Danhong injection; PCI, percutaneous transluminal coronary intervention; DAAT, double anti-platelet aggregation; PS, plaque stabilization (atorvastatin calcium tablets, etc.); a-gent, antihypertensive; UFH, unfractionated heparin; CE, coronary enlargement; MACEs, major adverse cardiovascular events; STR, ST segment resolution; TIMI, thrombolysis in myocardial infarction; CRP, C-reactive protein; hs-CRP, hypersensitive C-reactive protein; LVEF, left ventricular ejection fraction; CD62P, P-selectin CD62P; TC, total cholesterol; TG, triglyceride; LDL, low-density lipoprotein; ET-1, endothelin-1; Fg, fibrinogen; GP, glucose protein; FIB-C, fibrinogen C; sP-sel, soluble P-selectin; IL, interleukin; SOD, superoxide dismutase; Vwf, von Willebrand factor; sICAM-1, intercellular adhesion molecule-1; BNP, brain natriuretic peptide; MDA, malonaldehyde; ISR: in-stent restenosis; FMD, flow-mediated dilation; NO, nitric oxide; VCAM-1, vascular cell adhesion molecule-1; MIS, myocardial infarction size; NTG, endothelial non-dependent vascular dilation reactions; LpPLA2, lipoprotein-associated phospholipase A2; MMP, matrix metalloproteinase; Cys-C, cystatin-C; Hcy, homocysteine; IMR, index of microcirculation resistance; TNF-α, tumor necrosis factor-α; ANGPTL4, angiopoietin-like4; Sst-2, soluble suppression of tumorigenicity 2.

**Figure 2:**
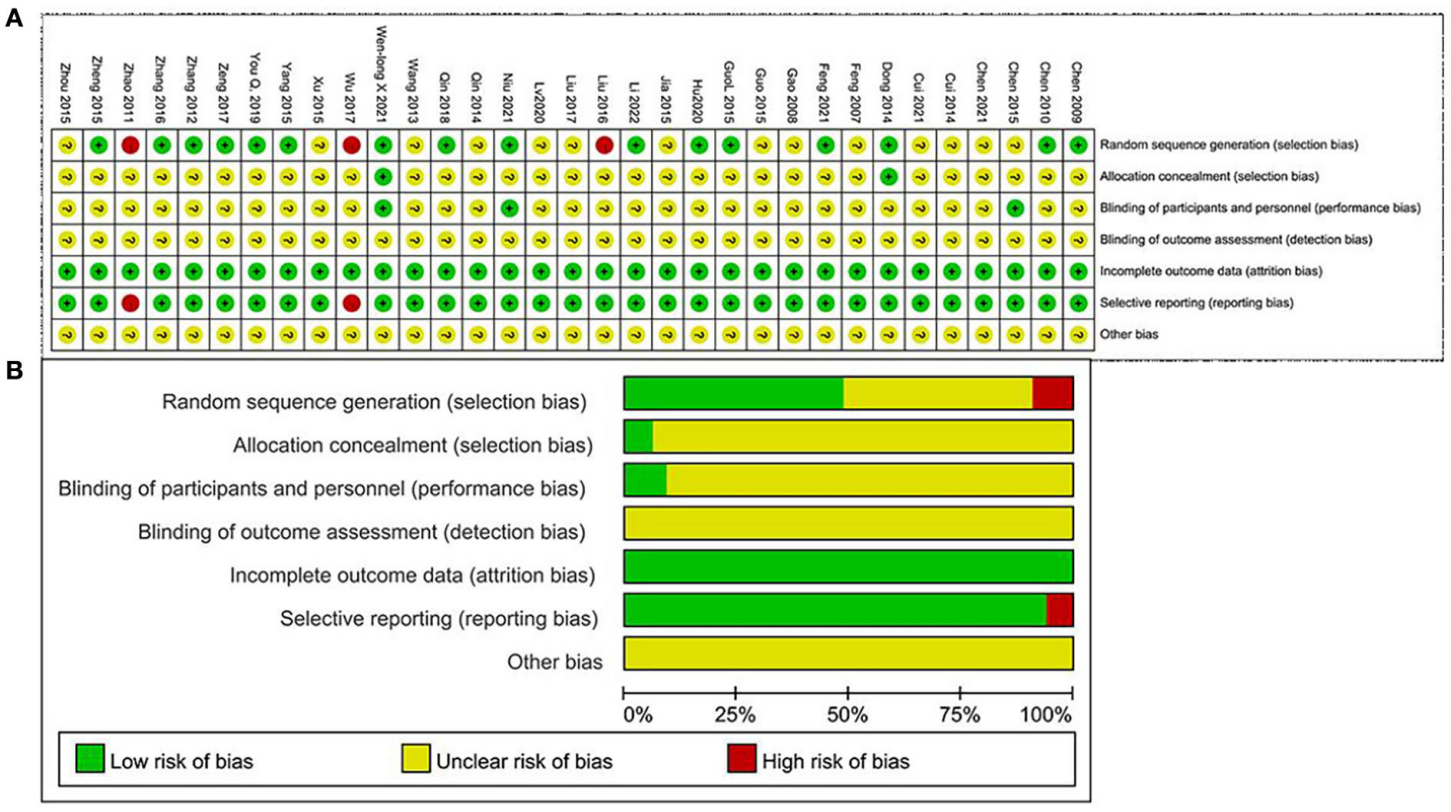
**(A)** Risk-of-bias summary. **(B)** Risk-of-bias graph.

The Cochrane Handbook tool was used for assessing the risks of bias in this study. In all the studies, subjects were randomly assigned. A total of 15 studies that provided the detailed information about that random assignment method had a low risk of bias; two studies that randomized subjects according to the time of admission and treatment protocol had a high risk of bias; eight studies that did not report information about the randomization method had an unclear risk of bias. Regarding allocation concealment, two studies that provided descriptions of allocation methods had a low risk of bias. The remaining 29 studies that did not describe the allocation process had an unclear risk of bias. Regarding blinding methods, two studies that blinded the patients but not the investigators had a low risk of bias. The remaining studies that did not report patient or investigator blinding had an unclear risk of bias. In addition, two studies that did not report the results of the pre-specified indicators had a high risk of bias due to selective outcome reporting. The remaining 31 studies that included complete data results had a low risk of bias. All included studies stated that the baseline characteristics of the two groups were not significantly different (Figure 2).

### Outcome measures and subgroup analyses

#### MACE

**Figure 3:**
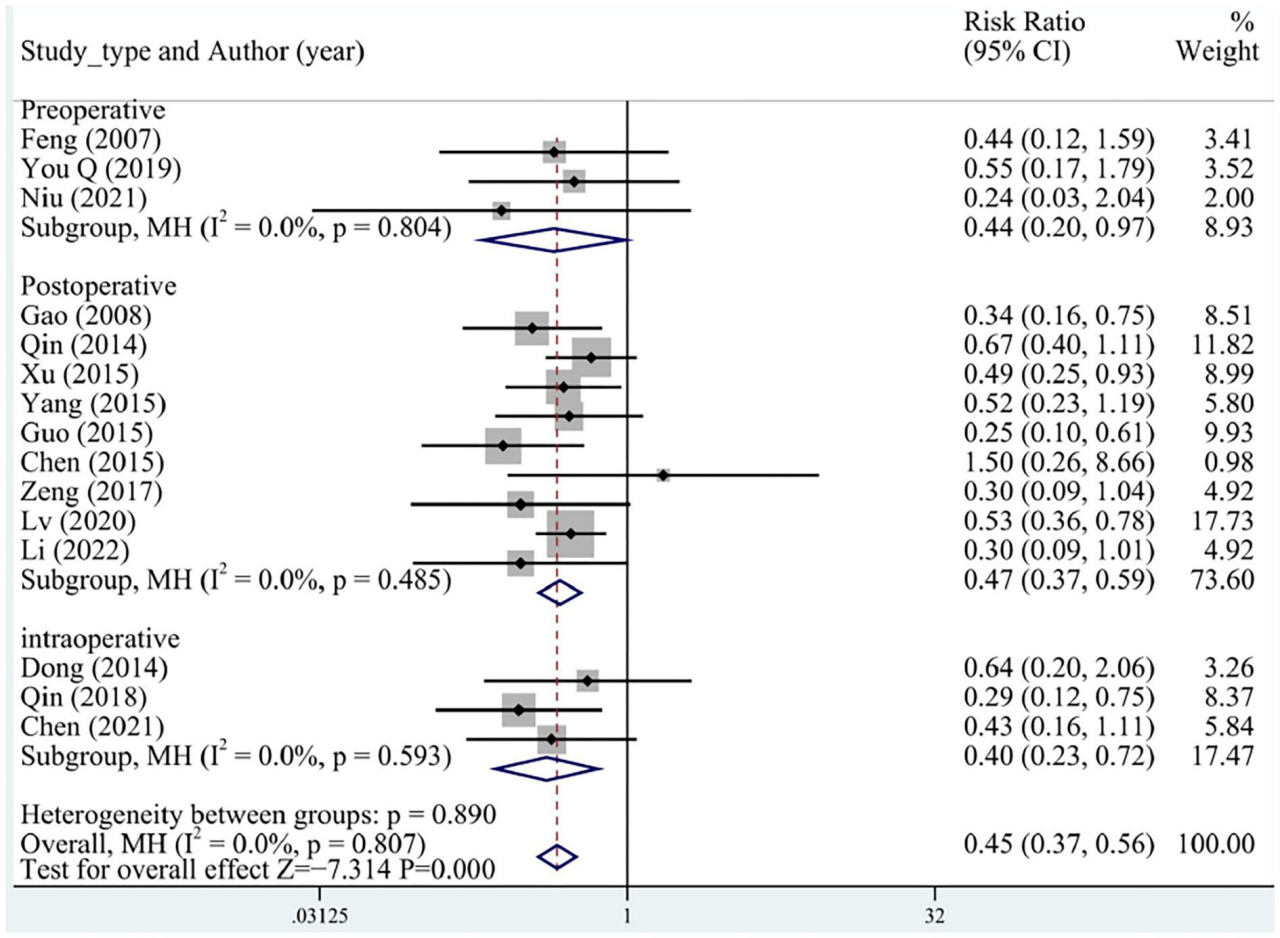
MACE subgroup analysis. MACE, major adverse cardiovascular event.

A total of 15 (three preoperative, three intraoperative, and nine postoperative) studies reported MACEs, including malign arrhythmias, angina pectoris, recurrent myocardial infarction, recurrent hemodialysis, heart failure, and cardiac death that occurred during follow-up. No significant heterogeneity was determined between the studies. Overall, the incidence of MACEs was significantly lower in the test group than in the control group (RR = 0.45, 95% CI [0.37, 0.56], *P* < 0.05, *I*^2^ = 0%), and no differences between preoperative, intraoperative, and postoperative groups were identified (Figure 3).

#### Reperfusion rate

The reperfusion rate was assessed using the postprocedural TIMI flow grade and STR. A TIMI ≥ grade 3 and an STR rate ≥ 50% indicated successful reperfusion.

**Figure 4:**
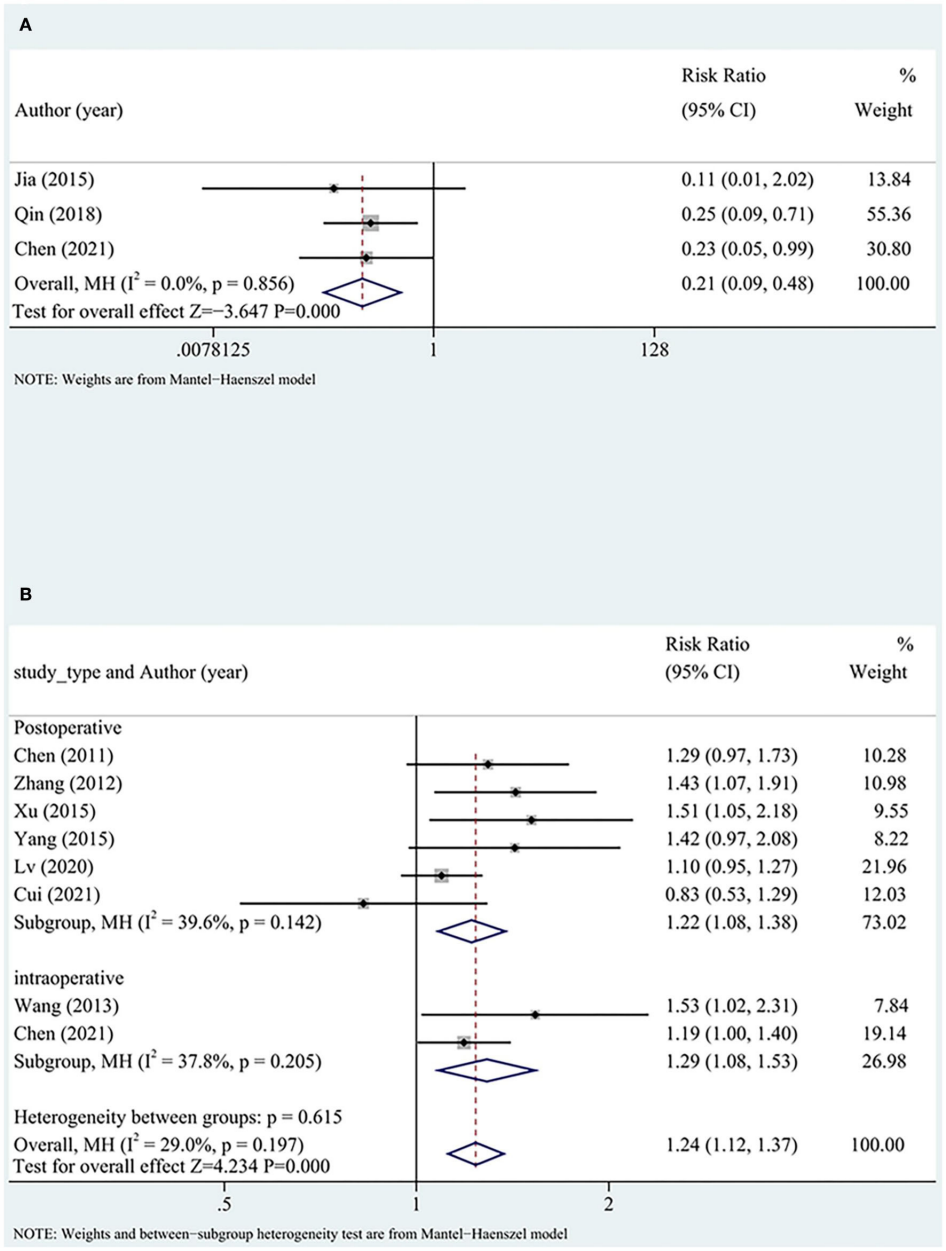
Reperfusion subgroup analysis including **(A)** TIMI flow grade and **(B)** STR. TIMI, thrombolysis in myocardial infarction; STR, ST segment resolution.

A total of three studies reported TIMI flow grades, and all studies applied DHI intraoperatively. Low heterogeneity was detected between the studies. The TIMI flow grade was significantly better in the test group than in the control group (RR = 0.22, 95% CI [0.10, 0.50], *P* < 0.05, *I*^2^ = 0%), suggesting that DHI could improve the TIMI flow grade of patients (Figure 4A).

Overall nine (three intraoperative and six postoperative) studies with high heterogeneity reported STR. STR was significantly better in the test group than in the control group (RR = 1.33, 95% CI [1.13, 1.58], *P* < 0.05, *I*^2^ = 66%). After excluding one study (32), the heterogeneity significantly reduced, although the findings did not significantly change (RR = 1.24, 95% CI [1.12, 1.37], *P* < 0.05, *I*^2^ = 29%).

The comparison of intraoperative and postoperative indicators between the two groups showed no differences. The reperfusion rate was more favorable in the test group than in the control group. The timing of DHI (intraoperatively or postoperatively) did not significantly affect the reperfusion rate (Figure 4B).

#### Inflammatory factors

**Figure 5:**
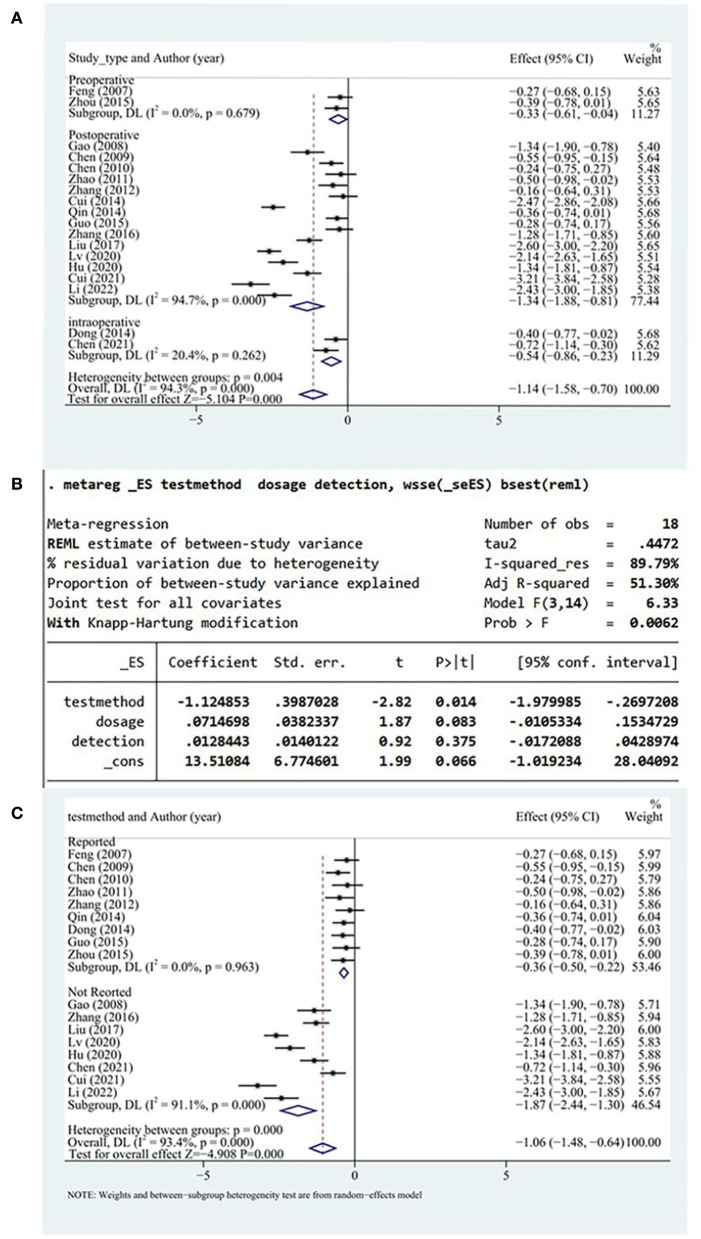
**(A)** hs-CRP subgroup analysis. **(B)** Results of meta-regression. **(C)** Subgroup analysis of test methods. Hs-CRP; high sensitivity C-reactive protein.

A total of 18 studies reported serum hs-CRP levels. Random-effects models were applied owing to the high heterogenicity. The serum hs-CRP levels more significantly decreased in the test group than in the control group (SMD = −1.14, 95% CI [−1.58, −0.7], *P* < 0.05, *I*^2^ = 94.2%). The hs-CRP level more significantly decreased when DHI was administered postoperatively than pre- or intraoperatively (Figure 5A). A meta-regression was conducted to identify the possible sources of the high heterogeneity, and the hs-CRP test method was identified as a source of heterogeneity (*P* < 0.05; Figure 5B). According to the test method, a subgroup analysis was undertaken based on the explicit test method. We excluded one study (22) to reduce the heterogeneity of the explicit test method groups. Subsequently, there was no heterogeneity in the explicit test method groups. The use of the unspecified assay method to measure the hs-CRP level was identified as a specific potential source of heterogeneity (Figure 5C).

**Figure 6:**
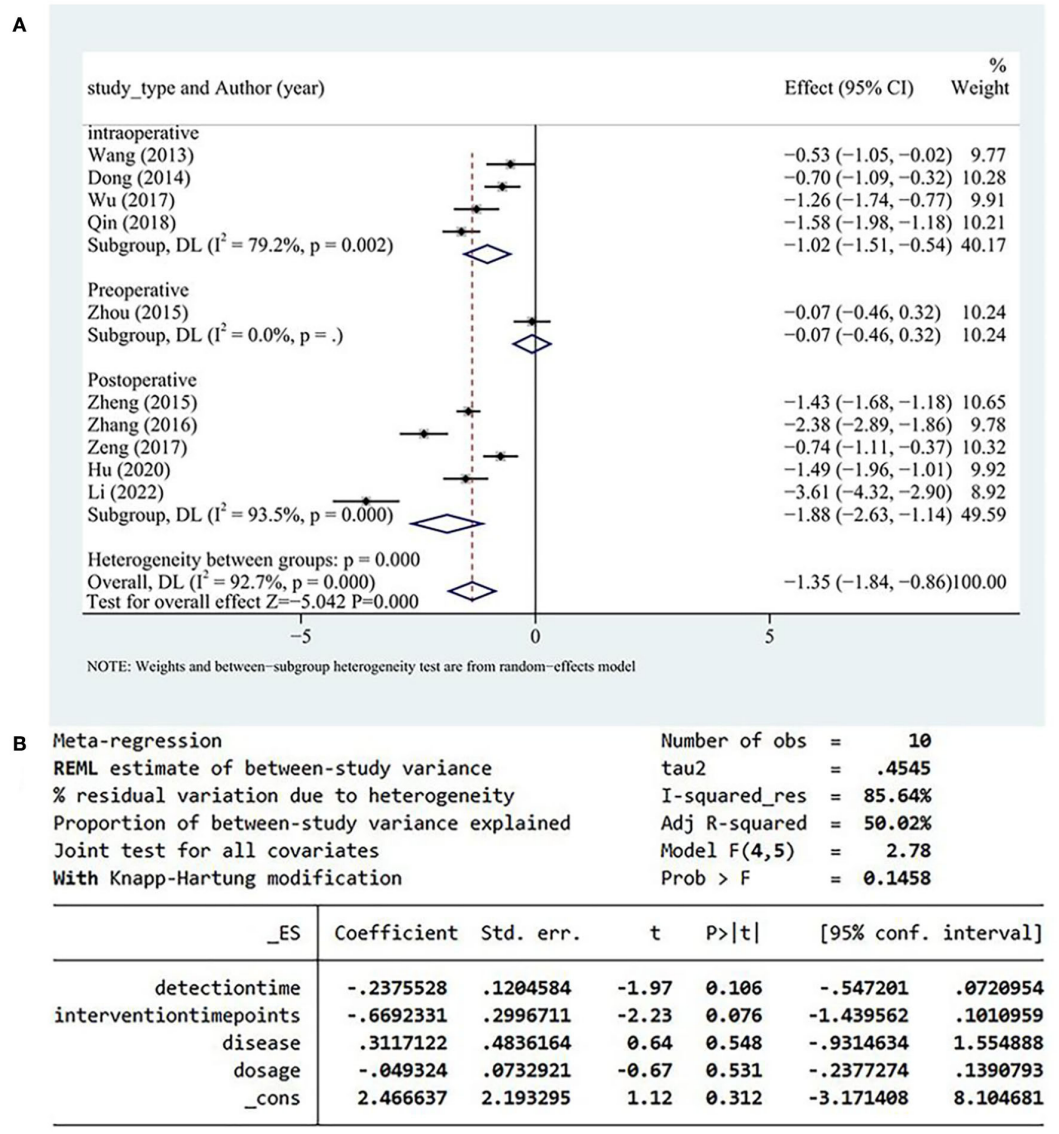
**(A)** IL-6 subgroup analysis. **(B)** Results of meta-regression. IL, interleukin.

A total of 10 studies reported serum IL-6 levels, and high heterogeneity was detected among these studies. Serum IL-6 levels more significantly decreased in the test group than in the control group (SMD = −1.35, 95% CI [−1.84, −086], *P* < 0.05, *I*^2^ = 92.7%; Figure 6A). A meta-regression analysis determined that the heterogeneity was independent of disease type, detection time, and drug dose and was correlated with the timing of DHI (Figure 6B).

Heterogeneity within the data of the intraoperative group originated from one study (34). After excluding that study (34), heterogeneity of the intraoperative group decreased from *I*^2^ = 79.2% to *I*^2^ = 54%. But the source of heterogeneity in the postoperative group was not identified after the separate exclusion of each study; therefore, the postoperative group was identified as the main source of heterogeneity.

#### Myocardial injury index

**Figure 7:**
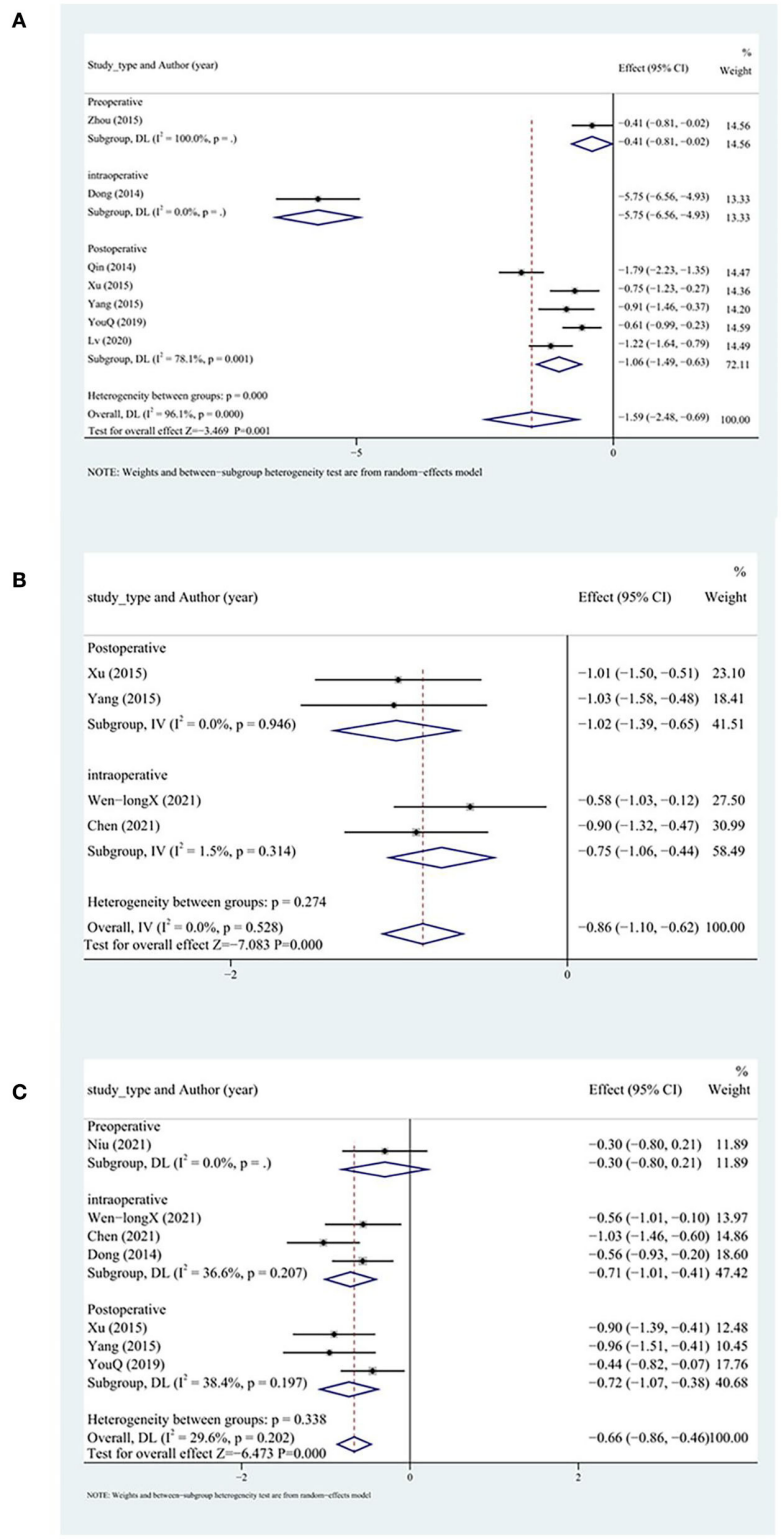
Myocardial injury index subgroup analysis including **(A)** cTnT, **(B)** CK, and **(C)** CK-MB. cTnT, cardiac troponin T; CK, creatine kinase.

In all, seven studies reported serum cTnT levels, five of which included patients with acute myocardial infarction in whom DHI was administered postoperatively, and the remaining two studies involved patients with unstable angina for whom DHI was administered preoperatively or intraoperatively. Random-effects models were applied because of high heterogeneity. The results indicated that the peak cTnT level more significantly decreased in the test group than in the control group (SMD = −1.59, 95% CI [−2.48, −0.69], *P* < 0.05, *I*^2^ = 96%). We removed one study (9) after sensitivity analysis with no significant heterogeneity within the subgroups. The results did not change significantly (SMD = −1.56, 95% CI [−2.60, −0.52], *P*<0.05, *I*^2^ = 96.5%; Figure 7A). Among the three subgroups, the intraoperative group had a significantly higher cTnT peak level.

A total of four studies reported CK levels. Fixed-effects models were applied because of low heterogeneity. The peak CK level more significantly decreased in the test group than in the control group (SMD = −0.86, 95% CI [−1.10, −0.62], *P* < 0.05, *I*^2^ = 0%; Figure 7B).

A total of nine studies reported CK-MB levels. Random-effects models were applied because of high heterogeneity. The peak CK-MB level more significantly decreased in the test group than in the control group (SMD = −1.05, 95% CI [−1.55, −0.55], *P* < 0.05, *I*^2^ = 90.6%). We searched for the source of heterogeneity by conducting a sensitivity analysis. After excluding two studies (9, 37), the heterogeneity decreased significantly. The peak CK-MB level more significantly decreased in the test group than in the control group (SMD = −0.66, 95% CI [−0.86, −0.46], *P* < 0.05, *I*^2^ = 29.6%). No differences between the intraoperative and postoperative groups were observed (Figure 7C).

#### Cardiac function

**Figure 8:**
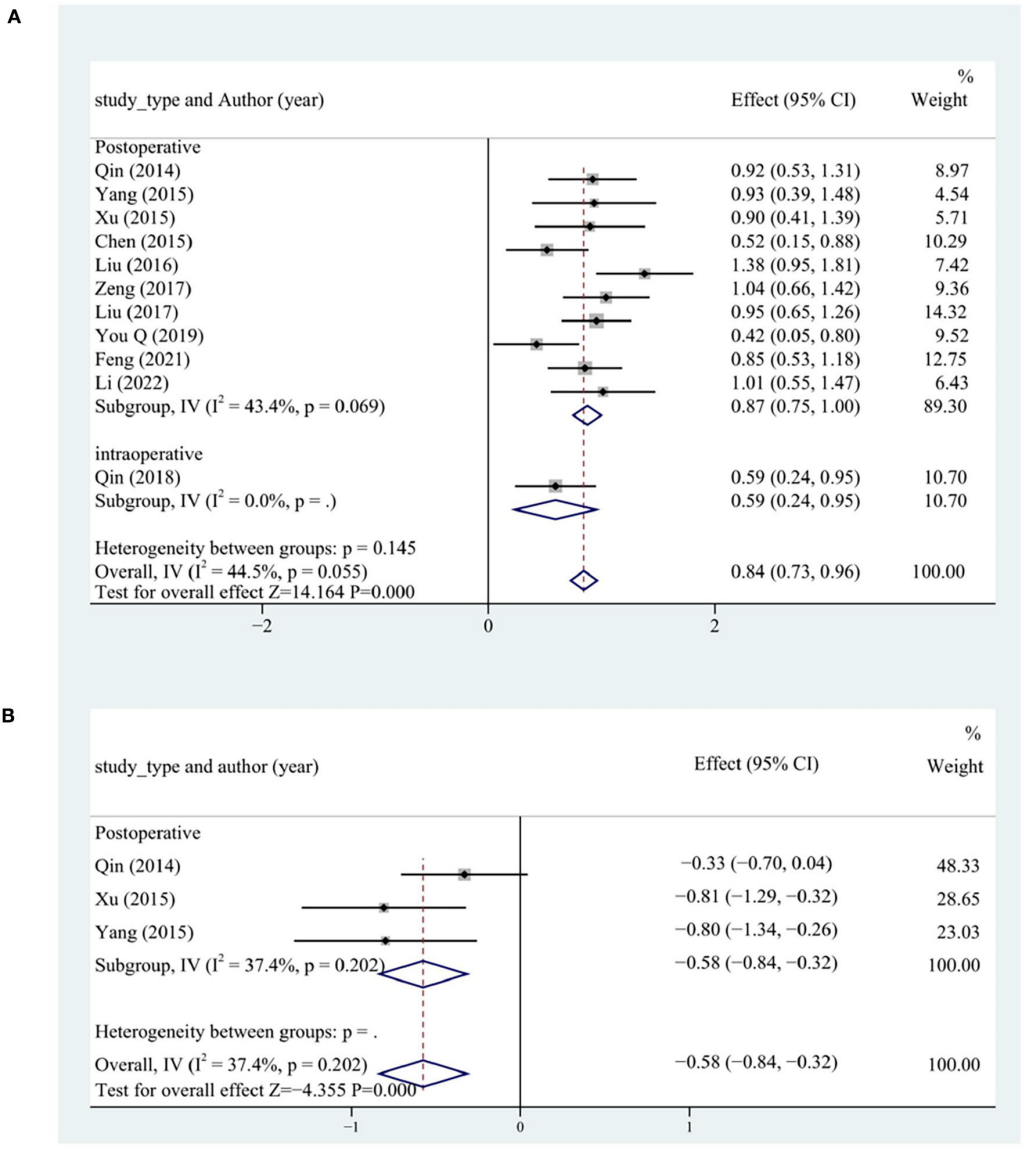
Cardiac function subgroup analysis including **(A)** LVEF and **(B)** BNP. LVEF, left ventricular ejection fraction; BNP, B-type natriuretic peptide.

LVEF and BNP levels were analyzed to assess cardiac function. A total of 12 studies reported LVEF levels. LVEF levels more significantly increased in the test group than in the control group (SMD = 0.96, 95% CI [0.68, 1.25], *P* < 0.05, *I*^2^ = 85.1%). The source of heterogeneity was determined by conducting a sensitivity analysis, and one study was identified as the main source of heterogeneity (27). After excluding that study (27), each subgroup had significantly lower heterogeneity. LVEF levels more significantly increased in the test group than in the control group (SMD = 0.84, 95% CI [0.73, 0.96], *P* < 0.05, *I*^2^ = 44.5%). The postoperative group had the most significantly increased LVEF levels (*P* < 0.05; Figure 8A).

In all, four studies reported BNP levels. The BNP levels more significantly decreased in the test group than in the control group (SMD = −1.96, 95% CI [−3.70, −0.21], *P* < 0.05, *I*^2^ = 97.7%). We searched for the source of heterogeneity by conducting a sensitivity analysis. After the exclusion of the study (29), the heterogeneity was significantly lower in each subgroup. The BNP levels more significantly decreased in the test group than in the control group (SMD = −0.58, 95% CI [−0.84, −0.32], *P* < 0.05, *I*^2^ = 37.4%). The postoperative group had the most significantly decreased BNP levels (Figure 8B).

### Publication bias

**Figure 9:**
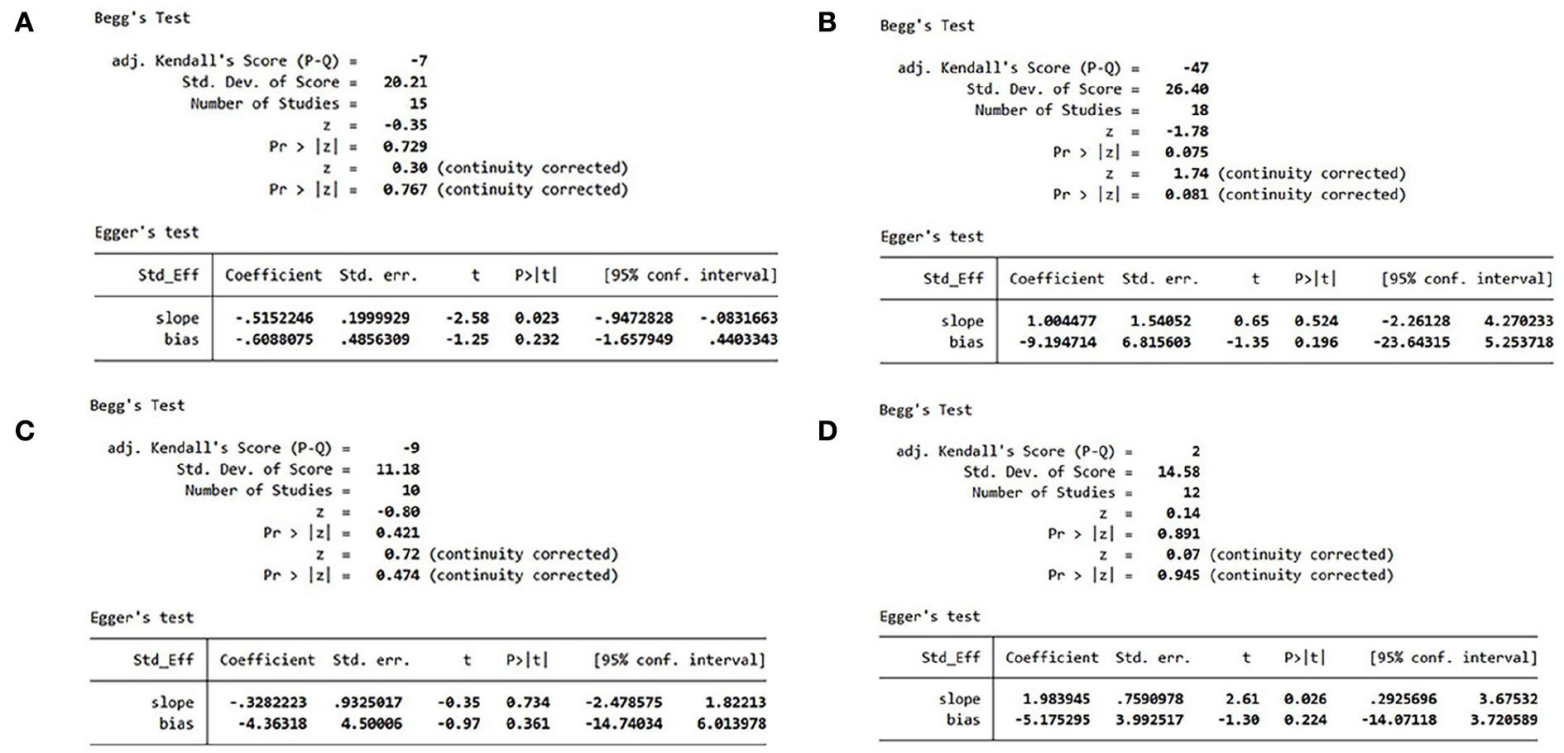
**(A)** MACE publication bias analysis. **(B)** hs-CRP publication bias analysis. **(C)** IL-6 publication bias analysis. **(D)** LVEF publication bias analysis. MACE, major adverse cardiovascular event; hs-CRP, high-sensitivity C-reactive protein; IL, interleukin; LVEF, left ventricular ejection fraction.

Since more than 10 studies reported MACEs, hs-CRP, IL-6, and LVEF, we performed Egger's and Begg's tests to identify publication bias for these studies. The results showed that there was no possibility of publication bias (Figure 9).

## Discussion

### Overview of evidence

A total of 33 studies, including four in which DHI was administered preoperatively, seven in which DHI was administered intraoperatively, and 22 in which DHI was administered postoperatively. In our study, 3,458 patients were included in meta-analysis. Data regarding the use of DHI in patients with ACS during the perioperative period of PCI were summarized. The combination of DHI and conventional treatment effectively decreased the number of inflammatory factors, the incidence of no reflow, myocardial injury, and the incidence of MACEs, and increased cardiac function. Postoperative DHI may result in more favorable suppression of the inflammatory response and improvement in cardiac function and patients' quality of life.

### Clinical application of DHI to ACS

The annual CHD incidence in China is increasing. ACS is the most serious type of CHD, and PCI is an effective treatment for ACS (3). PCI may be complicated by no reflow, arrhythmias, and MIRI. MIRI impairs cardiac function and negatively affects prognosis in patients undergoing cardiac surgery (44); there is a high risk of MIRI even when patients are administered conventional drug therapy, and MIRI contributes to up to 50% of the final infarct size (45). Therefore, the development of a method for attenuating myocardial injury to increase PCI efficacy is necessary.

In China, DHI is recommended for patients with ACS and patients undergoing PCI (46, 47). DHI is widely used in clinical practice and includes phenolic acid, C-glycosyl quinone chalcones, flavonoid glycosides, cyclic enol ether terpene glycosides, organic acids, amino acids, and nucleosides (46).

Several clinical studies had reported that the combination of DHI and conventional therapy may result in more favorable clinical outcomes than standard therapy alone. This meta-analysis also clarifies the clinical efficacy of DHI. Moreover, subgroup analyses are conducted to determine the optimal timing of DHI. Angina pectoris, heart failure, severe arrhythmia, recurrent myocardial infarction, re-bleeding, and cardiogenic death during follow-up are some of the MACEs. MACE is a reflection of the prognosis of patients; therefore, MACE is a primary endpoint in this study.

DHI improves patients' prognosis. When combined with conventional therapy, DHI decreases the incidence of MACEs. MIRI leads to adverse ventricular remodeling, resulting in progressive heart failure and poor outcomes. The results of the current meta-analysis indicate that DHI ameliorates cardiac function. After treatment with DHI, the LVEF level significantly improved (*P* < 0.05), and the BNP level significantly reduced (*P* < 0.05).

As a TCM standardized product, DHI has several targets and multiple effects. Hence, the mechanism of action of DHI remains unclear. DHI improves the reperfusion rate (*P* < 0.05) and decreases the CK, CK-MB, and cTnT levels in patients with ACS during the perioperative period of PCI (*P* < 0.05), indicating that DHI decreases the incidence of myocardial injury by improving the reperfusion rate. This may be the mechanism of action of DHI to increase cardiac function and improve patients' prognosis.

Myocardial ischemic injury and MIRI are associated with inflammation. Previous clinical studies have examined whether DHI exerts a protective effect by suppressing the inflammatory response. In the current meta-analysis, IL-6 and hs-CRP are used as indicators of pro-inflammatory responses. IL-6 induces inflammatory cell adhesion and injures vascular endothelium, and hs-CRP is a predictor of cardiovascular events. DHI more significantly decreases hs-CRP and IL-6 levels than standard therapy (*P* < 0.05). The mechanism of action of DHI in inhibiting inflammation may be multi-faceted. It is reported that DHI reduces inflammatory cytokines, such as IL-1, IL-18, MMP-9, and TNF-α, resulting in a broad anti-inflammatory effect (7, 27, 30, 32, 39, 43). Inhibition of inflammation by DHI is an important mechanism for its cardioprotective effect.

Although DHI is recommended for patients with ACS or patients undergoing PCI, the timing of DHI has not optimized yet. Patients included in this meta-analysis were divided into preoperative, intraoperative, and postoperative subgroups. The incidence of MACEs, TIMI flow grade, and STR was not significantly different between the subgroups. However, hs-CRP and IL-6 levels reduced more significantly in patients in whom DHI was administered postoperatively. The peak cTnT level was significantly decreased in the intraoperative group than in the other subgroups. In the postoperative group, the LVEF level was significantly improved (*P* < 0.05), and the BNP level was significantly reduced.

The results of this study suggest that DHI is effective in patients with ACS. While the incidence of MACEs is not affected by the timing of DHI, postoperative DHI may suppress the inflammatory response and improve cardiac function more significantly. Therefore, postoperative DHI is recommended to optimizing the efficacy of conventional treatment. However, the potential adverse effects of DHI must be considered. The incidence of adverse reactions of DHI is 3.50 per 1,000, and common adverse reactions include pruritus, rash, sweating, dizziness, and headache. Severe adverse effects such as anaphylactic shock are very rare (48). Current clinical studies do not report adequate data regarding the adverse effects of DHI. We suggest all clinical studies in progress to report adverse effects.

### Anti-inflammatory effects of DHI

DHI enhances cardiac function by inhibiting the inflammatory response, increasing the reperfusion rate, alleviating myocardial injuries, and ultimately reducing the incidence of MACEs. Inflammatory response plays an important role in MACEs and is closely related to ischemia–reperfusion injury. DHI alleviates myocardial injuries *via* anti-inflammatory effects exerted by multi-target pathways.

**Figure 10:**
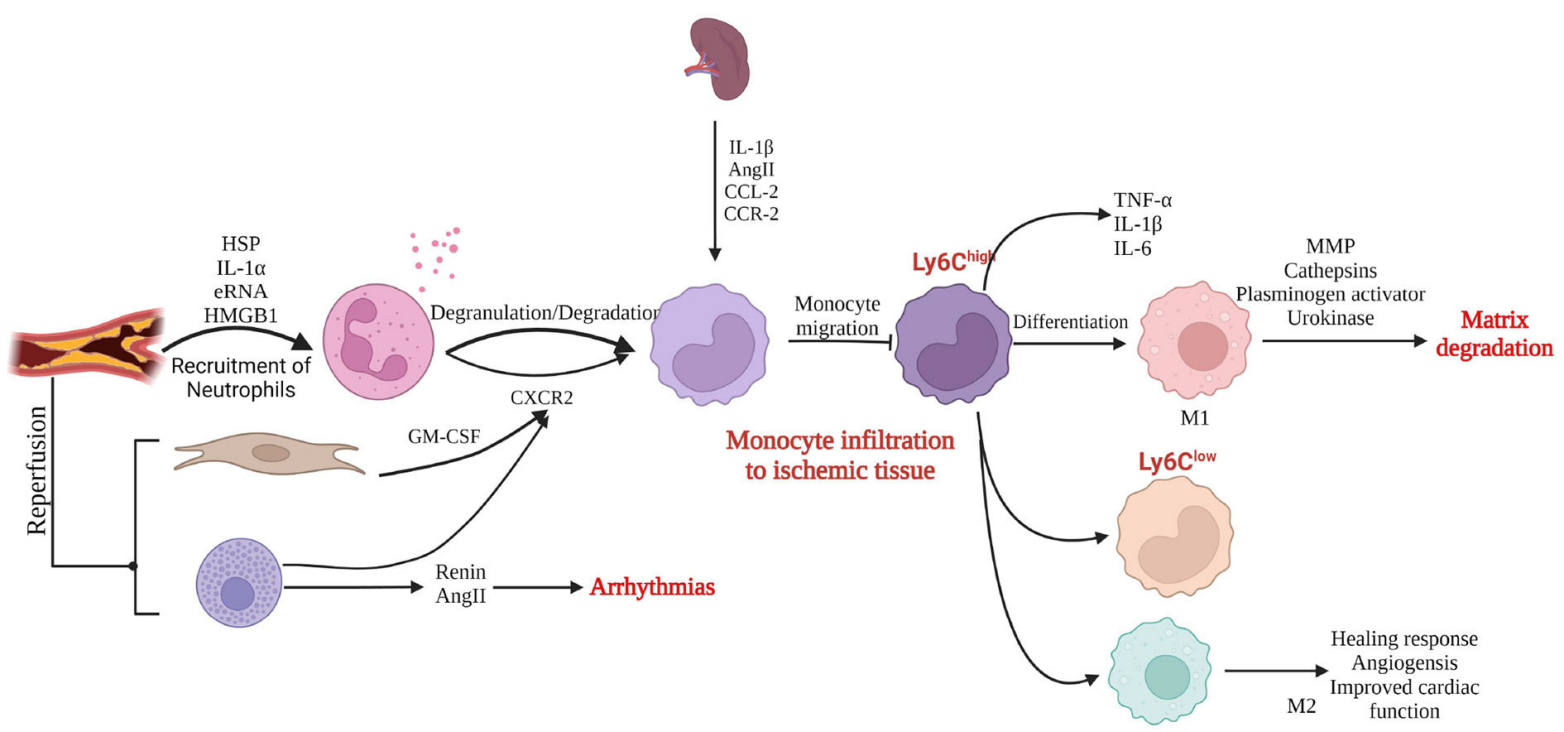
Inflammatory response to myocardial injury (created with BioRender.com). HSP, heat shock protein; IL, interleukin, RNA, ribonucleic acid; HMGB1, high mobility group box 1; GM-CSF, granulocyte–macrophage colony-stimulating factor; AngII, angiotensin II; CXCR2, CXC chemokine receptor 2; CCL-2, C-C chemokine ligand 2; CCR-2, C-C chemokine receptor type 2; TNF, tumor necrosis factor; MMP, matrix metalloproteinase.

Shortly after myocardial ischemic injury, necrotic cardiomyocytes release alarmins to activate the immune system and trigger neutrophil infiltration in the ischemic necrosis area (49). Neutrophils generate pro-inflammatory responses that trigger the infiltration of monocytes (50). When reperfusion is performed at this time, fibroblasts release granulocyte–macrophage colony-stimulating factor to promote neutrophil and monocyte infiltration in the ischemic necrotic area (51). Activation or degranulation of mast cells results in the release of pro-inflammatory mediators, and the derived angiotensin II (AngII) induces reperfusion arrhythmias by activating the renin–angiotensin system (52, 53). Few hours to days after myocardial ischemic injury, the composition of immune cells changes, and the spleen becomes a major source of monocytes. Monocyte migration to sites of myocardial injury is regulated by IL-1β, AngII, and the binding of chemokine ligand 2 and chemokine receptor 2. The first monocytes to migrate to the site of myocardial injury are pro-inflammatory Ly6C^high^ monocytes, which differentiate into activated pro-inflammatory macrophages; express IL-1β, IL-6, TNF-α, and protein hydrolases; and secrete MMPs, which degrade the extracellular matrix (54–57). At a later stage, both Ly-6C^low^ monocytes and the M2 phenotype are involved in angiogenesis and collagen deposition, forming scar tissue to replace lost cardiomyocytes in areas of ischemic necrosis and promoting the healing response of the ischemic myocardium (Figure 10).

The sustained and severe pro-inflammatory response during this process leads to adverse ventricular remodeling. Inflammation is an important novel target to ameliorate the prognosis of patients after PCI. Postoperative DHI better inhibits the inflammatory response and increases cardiac function, which may be related to adverse ventricular remodeling caused by the inhibition of the inflammatory response by DHI.

The results of this study indicate that DHI significantly reduces the hs-CRP and IL-6 levels (*P* < 0.05). IL-6 induces inflammatory cell adhesion and injures vascular endothelium, and hs-CRP is a predictor of cardiovascular events. In addition, DHI reduces the TNF level in patients with ACS (39, 43), leading to reduced expression of chemoattractant protein-1 in monocytes to inhibit inflammatory responses (58). Indicators of myocardial injury are further reduced after decreased inflammation in patients with ACS, and cardiac function is significantly improved. Cardiac function is closely related to the prognosis of patients, and the improvement of cardiac function reduces the incidence of MACEs.

### Strengths and limitations

A previous meta-analysis reported that DHI improved the total efficacy rate, reduced the inflammatory response, and inhibited oxidative stress in patients with ACS (59). Liao et al. reported that DHI reduced mortality and the incidence of MACEs, which were considered to be associated with improved cardiac function and reperfusion, in patients with acute myocardial infarction (60). Zou et al. conducted a meta-analysis of the effects of DHI in patients with ACS undergoing interventional procedures and showed that DHI improved the overall response rate of treatment and reduced the incidence of MACEs (11). However, no previous study evaluated the effects of the timing of DHI.

This study is a state-of-the-art study involving 3,458 patients and evaluates the clinical effects of the combination of DHI and conventional therapy in patients with ACS. Also, this study clarifies the effectiveness of DHI during the perioperatively period of PCI in patients with ACS, proposes a possible mechanism of action, and assesses the timing of DHI.

This study also has several limitations. First, the included studies were all conducted in China. To generalize these results to other populations, multi-national investigations should be conducted in future. Second, some included studies achieved low scores on quality assessment. Thus, future trials should be designed to meet the CONSORT criteria. Third, the number of studies regarding preoperative/intraoperative DHI was low, limiting the strength of the conclusions, which should be interpreted carefully.

## Conclusion

The combination of DHI and conventional therapy results showed a better therapeutic effect than conventional therapy alone in patients with ACS undergoing PCI. DHI decreases the incidence of MACEs and improves the reperfusion rate. DHI has multiple effects, including reducing inflammation, reducing myocardial injury, and adjusting cardiac function to play a cardioprotective role. DHI could be a useful supplement to perioperative PCI for patients with ACS. Therefore, postoperative DHI is recommended as a standard treatment for patients with ACS.

## Data availability statement

The original contributions presented in the study are included in the article/supplementary material, further inquiries can be directed to the corresponding author.

## Author contributions

YuL and DL carried out the protocol and drafted the article. DL and WW assisted in data collection, quality control, and project administration. WW and XL assisted with analysis methods. XL, PL, and YZ were involved in data management and analysis. YaL and QL designed and managed this protocol. All authors contributed to the final version of the article.

## Funding

This work was supported by the Capital's Funds for Health Improvement and Research (2020-2-4201, 2020-4-4204) and the National Natural Science Foundation of China (81973622).

## Conflict of interest

The authors declare that the research was conducted in the absence of any commercial or financial relationships that could be construed as a potential conflict of interest.

## Publisher's note

All claims expressed in this article are solely those of the authors and do not necessarily represent those of their affiliated organizations, or those of the publisher, the editors and the reviewers. Any product that may be evaluated in this article, or claim that may be made by its manufacturer, is not guaranteed or endorsed by the publisher.
